# Exploiting temporal and nonstationary features in breathing sound analysis for multiple obstructive sleep apnea severity classification

**DOI:** 10.1186/s12938-016-0306-7

**Published:** 2017-01-07

**Authors:** Jaepil Kim, Taehoon Kim, Donmoon Lee, Jeong-Whun Kim, Kyogu Lee

**Affiliations:** 1 Graduate School of Convergence, Science and Technology, Seoul National University, 1 Gwanak-ro, Seoul, 08826 Republic of Korea; 2 Department of Otorhinolaryngology, Seoul National University Bundang Hospital, Gumi-ro, Seongnam, 13620 Republic of Korea

**Keywords:** Obstructive sleep apnea, Breathing sound, OSA severity classification, Transition probability, Cyclostationary, Apnea-hypopnea index

## Abstract

**Background:**

Polysomnography (PSG) is the gold standard test for obstructive sleep apnea (OSA), but it incurs high costs, requires inconvenient measurements, and is limited by a one-night test. Thus, a repetitive OSA screening test using affordable data would be effective both for patients interested in their own OSA risk and in-hospital PSG. The purpose of this research was to develop a four-OSA severity classification model using a patient’s breathing sounds.

**Methods:**

Breathing sounds were recorded from 83 subjects during a PSG test. There was no exclusive experimental protocol or additional recording instruments use throughout the sound recording procedure. Based on the Apnea-Hypopnea Index (AHI), which indicates the severity of sleep apnea, the subjects’ sound data were divided into four-OSA severity classes. From the individual sound data, we proposed two novel methods which were not attempted in previous OSA severity classification studies. First, the total transition probability of approximated sound energy in time series, and second, the statistical properties derived from the dimension-reduced cyclic spectral density. In addition, feature selection was conducted to achieve better results with a more relevant subset of features. Then, the classification model was trained using support vector machines and evaluated using leave-one-out cross-validation.

**Results:**

The overall results show that our classification model is better than existing multiple OSA severity classification method using breathing sounds. The proposed method demonstrated 79.52% accuracy for the four-class classification task. Additionally, it demonstrated 98.0% sensitivity, 75.0% specificity, and 92.78% accuracy for OSA subject detection classification with AHI threshold 5.

**Conclusions:**

The results show that our proposed method can be used as part of an OSA screening test, which can provide the subject with detailed OSA severity results from only breathing sounds.

## Background

Obstructive sleep apnea (OSA) is the most common sleep-related breathing disorder. This syndrome is characterized by repetitive episodes of upper airway obstruction and commonly connected with a reduction in blood oxygen saturation. OSA is associated with a characteristic snoring pattern and consists of loud snores or short gasps that alternate with events of silence that typically last for 20–30 s.

Obstructive sleep apnea can induce various dangerous events or personal complaints in everyday life. For example, severe daytime sleepiness of patients caused by OSA could be a causative factor in a large number of motor vehicle accidents. Gastroesophageal reflux can occur as a result of the effort made to reestablish breathing. A loss of both libido and erectile ability could occur in patients with OSA. Cardiac arrhythmias also commonly occur during sleep in OSA patients. In this case, bradycardia alternates with tachycardia during the apneic phase and termination phase of the obstruction, respectively. Even more severe, tachyarrhythmias most commonly occur when a patient tries to reestablish breathing following the apneic phase and may increase the risk of sudden death during sleep.

Many population-based studies have reported a high prevalence of OSA in adults [1]. In the case of the United States, OSA has increased over the past two decades and its prevalence rate in adults between 30 and 70 years old has reached 26% [2]. Despite the seriousness and increased cases of OSA, related research has reported that 93% of women and 82% of men remain underdiagnosed [3]. The main reason for the high number of underdiagnosed individuals is that it is difficult to recognize the intensity of their pathological breathing during sleep. Even if they are aware of the symptoms, an expensive and uncomfortable examination prevents them from visiting hospital.

Polysomnography (PSG) is currently the gold standard for the diagnosis of OSA. To make an OSA severity diagnosis, PSG provides an Apnea-Hypopnea Index (AHI) that contains the number of apnea and hypopnea occurrences per hour of sleep. According to the American Association of Sleep Medicine (AASM), when a subject has more than five obstructive apneas over 10 s per hour of sleep, the individual could be suspected of having OSA syndrome [4]. However, the test should be conducted overnight and its cost is expensive. Moreover, the measurement is inconvenient because various physiological sensors must be attached to the body [5]. Because of these limitations of PSG, it is not suitable for mass examination and occasionally, obtaining reliable results is impossible because the patient has trouble sleeping. Recently, portable PSG has been developed and used in personal home care related to sleep disorders. However, this technology still requires multiple uncomfortable sensors and measurements for various physiological parameters, such as blood saturation and nasal airflow. Therefore, a preliminary screening test is necessary for suspected subjects who are concerned about the financial burden and measurement inconvenience. The test should be as simple as possible and should be capable of repeatedly measuring patient’s data for mass examinations.

Breathing sounds can be measured more easily than other known physiological signals during sleep. Conventional sensors can be used to take measurements in a body-contact manner, but the breathing signal can be recorded using non-contact sound recording devices. Moreover, most recent personal smartphones have a microphone that is sufficient to record an individual’s breathing sounds in the vicinity of the bed; thus; breathing sounds can be measured without any help from specialists or technicians. Moreover, many studies show that sleep breathing sounds are related to sleep disorders [6–13]. These studies could be representative examples of the medical advantages of being able to examine some symptoms related to sleep disorders without additional bio-signal sensors when high-quality sleep breathing sounds can be obtained from patients. Therefore, sleep breathing sounds can be regarded as acoustic physiological signals that can be measured by anyone.

However, most recent studies have focused on snoring segment detection, snore/non-snore classification, or OSA/non-OSA patient group classification. The sensitivity result of OSA classification, which has shown that a percentage of people with OSA are correctly identified as having the symptom, ranges from 60 to 80% in related studies [7, 8, 13]. For an efficient OSA screening test, OSA severity should be able to report results based on a clinical standard. According to the AASM, AHI values are categorized into four severity labels: normal, mild, moderate, and severe sleep apnea. Moreover, many studies have used body-contact microphones, for example, microphones attached to a surrounding area of the neck [6, 7, 12] or face [13]. These contact microphones easily cause inconvenience for patients and make it difficult to make simple measurements. Additionally, numerous studies have acquired breathing sounds using expensive professional microphones that typically hang from the ceiling at a short distance from the patient. More detailed information on the algorithms of previous studies is presented in a discussion section comparing the results of other studies with those of the proposed study.

The aim of this study is to develop a new approach of multiple OSA severity classification using breathing sounds during sleep. Two novel methods, the total transition probability of approximated sound energy in a time series and the statistical properties which are derived from dimension-reduced cyclic spectral density, are proposed for our object. To the best of our knowledge, so far, no approach has utilized a combined feature set, which was made with foregoing methods, for multiple OSA severity classification. In contrast to related studies [6, 7, 12, 13], breathing sounds are recorded using an ordinary microphone that is placed at a long distance from the patient and not intended to record special sounds, such as the patient’s breathing. Moreover, we focus on breathing sounds during non-rapid eye movement (NREM) sleep: stages 2 and 3 sleep. We know that sleep apnea-related snoring is most likely to occur during REM sleep. However, because we use an ordinary subject’s breathing sounds, we concentrate on the aforementioned two sleep stages in which conventional snoring is most likely to occur. Furthermore, body movement or other complex behaviors rarely occur during these stages, hence we can minimize the noise that is unrelated to breathing sounds. Additionally, we attempt to extract succinct characteristics from relatively long audio recordings without any particular event detection method or random event selection in contrast to previous studies [6, 7, 9, 11, 12]. The major contribution of this study is to find a new combined feature set of sleep breathing sounds for multiple OSA severity classification, which includes energy transition probability of audio signal and statistical data derived from cyclostationary analysis.

## Methods

To develop an OSA severity classification method, the recorded breathing sounds were divided into four OSA severity groups. All breathing sounds were acquired from PSG room monitoring video clips that were included in the clinical diagnostic tool. The spectral subtraction technique which is popular for the enhancement of noisy speech signal was applied in the preprocessing [14]. Then, the total transition probability of the approximated sound energy and the statistical properties of the modified cyclic spectral density features were extracted from the preprocessed breathing sounds. Using these features, we trained an OSA severity classification model using machine learning techniques and validated its accuracy. In this section, we first describe our participant subjects, physical recording environment, and sound acquisition method. Second, we explain the details of the two aforementioned feature extraction methods. Finally, we describe the training and validation method for the classification model.

### Breathing sound database

**Fig. 1:**
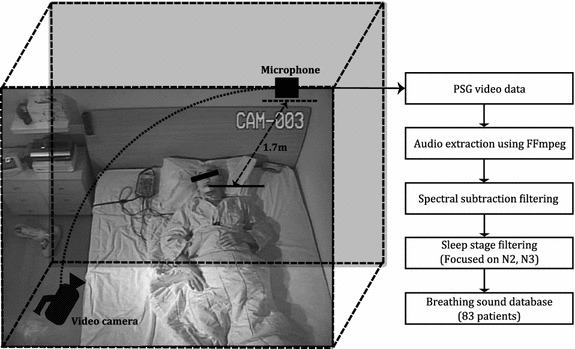
Sound acquisition and preprocessing in the PSG room. Audio data were extracted from the PSG monitoring video and then the two filtering methods were applied for 83 patients

A total of 83 adult subjects (27 females and 56 males with a mean age of 48.7 (±17.5) years, mean body mass index of 25.6 (±4.1), and mean AHI of 23.6 (±25.3)) were enrolled from the sleep laboratory of the Seoul National University Bundang Hospital (SNUBH), South Korea. The study was approved by the institutional review boards at SNUBH and informed consent was obtained from all patients or their guardians on their behalf. The PSG room contained a video camera and auxiliary microphone (SURP-102, YIANDA electronics Co., Ltd, ShenZhen, China; 20–2 kHz frequency range, −40 dB sensitivity) for monitoring the test for the entire night. The video clips were synchronized with various physiological signals of the PSG and stored using the sleep laboratory’s sleep diagnostic software (REM-Logic, Natus Medical Inc. CA, USA). The auxiliary microphone was located on the ceiling above the patient’s bed at a distance of 1.7 m. This microphone was originally installed in the PSG room and was not specially equipped for our experiments. Additionally, it was not special sound equipment intended for clinical purposes. Because the unimpressive, omnidirectional microphone was relatively far away from the patient, almost attached to the ceiling, various environmental sounds in the room were recorded together with breathing sounds. Since this recording environment has a poor SNR (signal-to-noise ration) of the audio signal than the general environment where the personal portable device is placed near the user’s sleep position (for example, around the head), we assumed that the sounds recorded in this study are similar to or worse than those recorded with personal devices that do not take into account the source location of the sound in the user’s general bedroom. The left-hand side of Fig. 1 shows the actual setup of the PSG room. From the video clips, the all-night breathing sounds of each subject were extracted using a multimedia converting tool (FFmpeg) [15] and saved as a wave format file with an 8 kHz sampling frequency. Then, according to each patient’s AHI value from the PSG test result, wave files were categorized into four OSA severity groups: normal (0 ≤ AHI ≤ 4), mild (5 ≤ AHI ≤ 14), moderate (15 ≤ AHI ≤ 29), and severe (AHI ≥ 30). The normal group included 20 breathing sounds and all other OSA groups contained 21 sounds. The average time of the breathing sounds was 7 h 10 min 30 s.

### Preprocessing

Since the microphone was not specifically chosen for sound analysis experiments, and was intended to monitor the PSG room’s test environment, there was a lot of background noise, such as white noise, hum or hiss when we checked the audio signals. We considered that these noises’ spectrums did not substantially change the target signal; thus, we adapted a spectral subtraction method [14], which is a computationally cheap and effective method for this situation. We assumed that subject’s ordinary breathing during sleep could be used to estimate OSA severity. When we checked the typical hypnograms of adults, stage 2 and 3 NREM sleep comprised most of the sleep, with a minimum of 60% [16]. During these sleep stages, the subject is typically stationary and the respiratory pattern is regular. Therefore, we assumed that regular breathing sounds could be obtained and various noise associated with the subject’s body movement and arousal could also be minimized during these stages. Thus, we focused on sleep breathing sounds during stages 2 and 3 NREM sleep and extracted this data from the original breathing sound database. Breathing sound data were simultaneously stored with physiological data from the PSG test and synchronized. Additionally, sleep stages were labeled within the clinical sleep diagnostic software by sleep specialists or physicians. Therefore, we could extract breathing sounds based on the time-stamped sleep stage information from the software. The average time of all extracted breathing sounds related to stages 2 and 3 NREM was 4 h 1 min 55 s (±1 h 34 min 59 s), which was significantly reduced sound data compared with the original sound database.

The right-hand side of Fig. 1 shows the preprocessing procedure used in the present study. The event detection method was not adapted to breathing sound analysis, in contrast to previous related studies [6, 7, 9, 11, 12]. This was to reduce possible errors and computational costs during the pre-detection process of targeted events. Additionally, our proposed method could use most respiratory sounds associated with normal breathing, snoring, and other disorders. The breathing sound was simply divided into window units of predetermined window length and sequentially entered into the following feature extraction methods.

### Feature extraction methods

We considered the two main features related to time and spectral domains. The time domain feature has the advantage of a great deal of information about the direct temporal characteristics of OSA and other breathing events. Additionally, the spectral domain feature provides good information because it represents the hidden properties of each sound unit; thus, it could identify the target data that could not be recognized through the time domain features. The first feature in this study is the total transition probability of approximated breathing sound energies in the time domain. The second feature is derived from the cyclostationarity-based information of breathing sounds, which presents hidden spectral characteristics using the periodicity of the signal’s autocorrelation. This was simplified and transformed into a statistical representation. All features were calculated using signal processing and statistical functions of MATLAB 2015a (MathWorks, Inc., MA, USA) on a Windows PC (Intel Xeon 3.3 GHz, 16 GB RAM, Windows 10 Pro). Next, we describe the details of the two feature extraction methods.

### Time domain analysis

The sequential property change of the PSG data in the time domain is the basic reference for diagnosing sleep apnea in a clinic, for example, an episode of more than 20 s respiratory arrest is an important indicator of apnea during sleep [4]. We assumed that as the frequency of obstructive sleep apnea increased, the frequency and length of silent intervals between breathing sounds would be increased, so that the sequential amplitude changes of breathing sounds during sleep could represent the incidence of obstructive sleep apnea. Using this characteristic, we summarized the signal transition information of the subject’s breathing sound as features for OSA severity classification. Using a 0.5-second Hanning window with 80% overlap, each subject’s breathing sound was segmented. Each segment was transformed into energy values and then approximated into three simple energy levels using thresholds (level 1: silence, level 2: lower energy level, and level 3: higher energy level).

Snoring has two dominant patterns of simple and complex waveforms. The complex-waveform snore is associated with palatal snoring and may represents actual airway obstruction through colliding of the airway walls. The simple-waveform snore does not actually obstruct the lumen, but it is generated by the oscillation around its neutral position and is associated with tongue-based snoring. Previous study shows that the palatal snoring has a higher ratio of peak sound amplitude to effective average sound amplitude than nonpalatal snoring [17]. Based on these facts, we assumed that level 2 could be represented energy level of general breathing events, including simple snoring, and level 3 included energy level of more louder snoring related to OSA events. Two dynamic thresholds were applied to divide the energy signal into two levels, that were sequentially updated from a predefined ratio of the most frequent energy peak range in each window segment. To eliminate some ripples, which were produced by an accumulation of the low energy values of the energy conversion process, we calculated another threshold, which was the proportion of actual signal energy within the area of a window. For instance, if the maximum energy value in a certain energy window was higher than the lower threshold and its energy proportion was more than 50%, this window was simplified as level 2. This window frame would only be level 1 if the energy proportion was lower than 50%. The threshold was applied to the early energy conversion process and reduced the errors of the energy approximation process.

During the second stage of this analysis, the OSA suspected sections were searched using the length of level 1 (silence) and the occurrence of other levels around level 1. When the level 1 section lasted for more than 20 s and was located between levels 2 and 3, this silent section was changed to level 4, which was an extra weight for the OSA suspected section; that is, level 4 particularly indicated that this section was an OSA candidate.

**Fig. 2:**
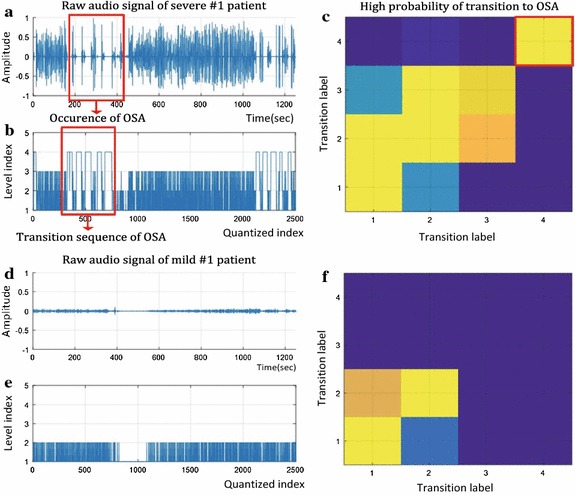
Representative example of the time domain analysis of sleep sound. **a** A raw audio data example of the OSA severe group and **b** its quantized signal’s energy. **c** The 4 × 4 transition matrix including probability values, which is calculated by (**b**). **d**–**f** Example results of the OSA mild group

During the third stage of analysis, the aforementioned approximated and weighted signal was transformed into a transition matrix. Because the signal had four levels, the matrix was 4 × 4 and 16 cases of the transition were accumulated as the matrix elements. By normalizing the matrix, the cumulative numbers in the elements were transformed to probability values, which represented the tendency of the subject’s breathing sound energy transition. As a result, these 16 probability values were features of the time domain analysis in this study. Figure 2 shows a representative example of this method.

### Nonstationary analysis based on cyclostationarity

We did not consider specific sound analysis based on event detection methods. Therefore, a window, the basic unit of analysis, included unspecific waveforms or noise. Furthermore, the dominant signal in a window was considered as a nonstationary signal, which included repetitive and complex waveforms such as breathing or snoring. Thus, characteristic properties representing not only the window’s basic properties but also an overall summary were required for our goal. In the case of snoring, it is the result of an obstruction of air flows in the respiratory tract during sleep and provokes repetitive vibrations of the tissues of the throat [18]. Therefore, the snoring sound could be considered as including two main types of waveform. The first is a complex waveform with a low frequency sound that is generated as a result of the collision of opposing airway walls during passing periods of airway obstruction. The second is a simple waveform sound with a quasi-sinusoidal pattern that could be considered as a result of the airway walls’ vibration around a neutral position without an obstruction of the respiratory tract lumen [19]. To obtain a valuable insight using the untapped reserves of the analysis method, we attempted to extract the cyclostationary properties from the sleep breathing sounds. It is possible to deduce that a signal is cyclostationary when it is nonstationary and its statistical characteristics vary periodically in the time domain [20]; that is, if the signals can decompose to the several sinusoidal wave components through a nonlinear transformation of order *n*, the signals are defined as an *n*-th order cyclostationary process. In the present study, an autocorrelation function, a second order statistic, was used for the nonlinear transformation of the signals. Therefore, if the second-order statistic of the signal was periodic, it was second-order cyclostationary. We first calculated a bivariate autocorrelation function *C*_*xx*_(*t,τ*) for each window of breathing sound *x*(*t*), with time(*t*) and time lag(*τ*). Then, we converted this function using a two-dimensional Fourier transform and the result was the spectral density. The spectral density *S*_*xx*_(*α,f*) must consist of two frequency variables: frequency *f* and cyclic frequency *α*. Only if *f* and *α* frequencies were related to some hidden frequencies was the spectral density *S*_*xx*_(*α,f*) continuous over *f,* while simultaneously a discrete function over *α* with non-zero values [21]. This non-zero spectral density, called the cyclic spectrum, and the spectral density *S*_*xx*_(*α,f*) are commonly known as the cyclic spectral density (CSD):

1 $$C_{xx} \left( {t,\tau } \right) = E\left[ {x \left(t + \frac{\tau }{2}\right)x^{*} \left(t - \frac{\tau }{2}\right)} \right] ,$$

where * denotes the complex conjugate,

2 $$S_{xx} \left( {\alpha ,f} \right) = \mathop {\lim }\limits_{T \to \infty } \frac{1}{T}\mathop \int \limits_{{\frac{ - T}{2}}}^{{\frac{T}{2}}} C_{xx} \left( {t,\tau } \right)e^{{ - j2\pi \left( {f\tau + \alpha t} \right)}} dtd\tau .$$

To derive the overall cyclostationary features of a full night’s sleep breathing sounds, the real-time mean of CSD, *rmS*_*xx*_(*α,f*), was calculated using the current CSD and a previous mean value for every 60-second window, where *rmS*_*xx*_(*α,f*) is described as follows:

3 $$rmS_{xx} \left( {\alpha ,f} \right)\left( k \right) = rmS_{xx} \left( {\alpha ,f} \right)\left( {k - 1} \right) + \frac{{S_{xx} \left( {\alpha ,f} \right)\left( k \right) - rmS_{xx} \left( {\alpha ,f} \right)\left( {k - 1} \right)}}{k}$$

where *k* = 1,2,3,*…,N* and *N* is the total number of windows. The number of steps in the *f* and *α* domains were 54 and 889, respectively. Therefore, *rmS*_*xx*_(*α,f*) generated a 54 × 889 matrix, with the magnitudes of *rmS*_*xx*_(*α,f*) as the elements. However, it was not sufficient to use the complete matrix of *rmS*_*xx*_(*α,f*) as a feature because of the widespread zero values. For dimensionality reduction, first, a threshold using Otsu’s method was applied to the *rmS*_*xx*_(*α,f*) matrix to eliminate unnecessary zero regions. Second, a non-negative matrix factorization (NMF) technique was applied to the previous matrix. NMF can analyze large quantities of data through the approximated decomposition of target data *V* into non-negative factors, which consist of a basis matrix *W* that includes inherent properties of data, and a coefficient or activation matrix *H* [22]. To obtain a representative basis matrix, a total of 83 previous *rmS*_*xx*_(*α,f*) matrices were sequentially merged into an input matrix and the basis matrix was calculated using NMF with 45 rank. The rank was heuristically determined by repetitive tests. This basis matrix was used as an initial basis matrix to calculate the activation matrix *H* of each *rmS*_*xx*_(*α,f*) matrix. Because a cyclostationary component-related basis matrix should be obtained, the transposed *rmS*_*xx*_(*α,f*) matrices were used in the aforementioned process, that is, the representative basis matrix contained inherent properties of cyclostationarity and we could extract a feature set consisting of the *H* matrices:

4 $$V \equiv WH$$

**Fig. 3:**
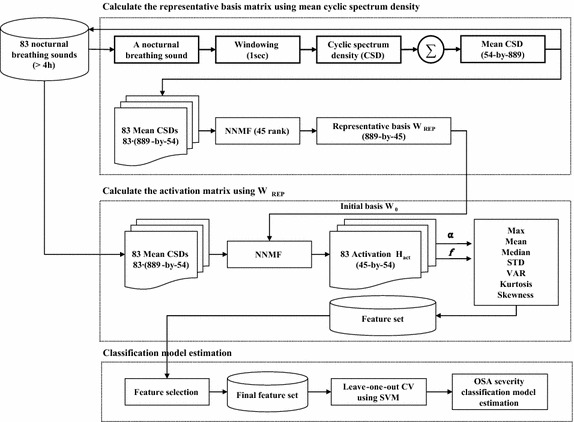
Feature extraction and classification based on nonstationary analysis. Statistical cyclostationary properties were extracted using the mean cyclic spectral density (CSD) and the non-negative matrix factorization (NMF) for dimension reduction

Because *WH* is an approximated matrix of *V*, the factors *W* and *H* were chosen using the minimization of the root mean squared (RMS) residual between *V* and *WH*. Through these procedures, *rmS*_*xx*_(*α,f*) (54 × 889) of a full night’s breathing sound was transformed into an NMF activation matrix (45 × 54). Based on the dimension-reduced matrix, we calculated seven basic statistics, maximum, minimum, median, standard deviation, variance, kurtosis, and skewness, according to the rows and columns of *H*. As a result, these 693 (45*7 + 54*7) statistical values are the features of nonstationary analysis based on cyclostationarity and we defined this statistical data set as the second feature in this study. This feature can analyze the statistics of the activity of the cyclic spectrum magnitude based on the spectral or cycle frequency domain. Figure 3 shows the second feature extraction process.

### Feature selection

For better classification performance, we conducted a feature selection process that eliminated redundant data in the aforementioned two features. We used the wrapper subset evaluation method, which is a flexible supervised attribute selector. We used a support vector machine (SVM) as a classifier of the evaluator and linear forward selection as an attribute search method. This process was performed using the WEKA framework [23], which embedded various attribute selection methods. Above-mentioned linear forward selection technique can find smaller optimal attribute subsets from full attributes and can reduce a risk of overfitting, finally it can provide higher classification accuracy [24]. This technique initially ranks all attributes and selects top-k ranked attributes by their scores that are obtained using a foregoing wrapper evaluator. Using limited number of attributes and m-fold cross-validation, this search technique finds the optimal subset size. Therefore, a result subset has an explicit size, and this is final feature set as input to classifier.

### Sleep breathing sound classification

Using the selected subset features, we performed three classification tests. For two types of features, we performed the individual classification test using related subset features. Then we conducted the final classification test with all subset features to validate the performance of multiple OSA severity classification.

All the classification tests provided the accuracy of the four OSA severity classifications using leave-one-out cross-validation (LOOCV). Additionally, we calculated the sensitivity specifically based on the four OSA severity classification results to compare classification performance. In this section, all tasks were conducted using the WEKA framework [23], which provides various feature selection methods and machine learning classifiers.

## Results

### Subset features for OSA severity classification

**Fig. 4:**
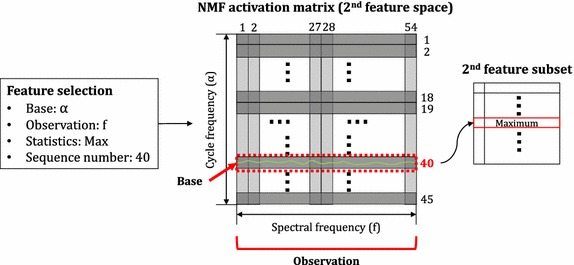
Feature selection from the NMF activation matrix. *x*-*axis* represents the spectral domain and the *y*-*axis* represents the dimension-reduced cycle frequency (α) domain. Based on this matrix, seven basic statistical values were calculated along each axis: α and f index

**Fig. 5:**
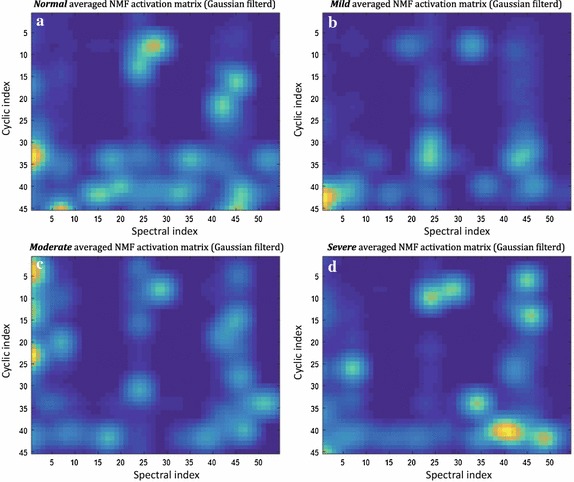
Averaged four NMF activation matrices based on the final nonstationary subset features. Dimension-reduced nonstationary features, the NMF activation matrices show different distributions of corresponding magnitudes for each dimension-reduced cycle frequency *α* and spectral *f* index pair: **a** normal **b** mild OSA, **c** moderate OSA, and **d** severe OSA

Using the feature selection method, we obtained 18 features from two types of features. Three were selected from the temporal analysis features and the remainder were selected from the nonstationary features. Table 1 shows the full subset feature list. In this table, we show the results for the rank, base, observation, statistics, and sequence number. “Rank” was determined using an attribute search method: wrapper subset evaluation feature selection method. A higher rank means that the associated feature is more significant for the classification task. In this study, the final nonstationary analysis feature space, called an NMF activation matrix, has a 45 × 54 dimension. Its x-axis represents the spectral domain and the y-axis represents the dimension-reduced cycle frequency (α) domain. Based on this matrix, seven basic statistical values were calculated according to each axis: *α* and *f* index. “Base” indicates a base axis for observing the statistical activation status of the other axis, which is presented in the “Observation” column. “Statistics” represents the type of statistics that were calculated for the observation axis. “Sequence number” represents an index of a particular base axis. For example, if the base is *α*, observation is *f*, statistics is maximum, and sequence number is 40, then we use a maximum of the *f* index domain’s activation statuses associated with the 40th index of the dimension-reduced cycle frequency index domain as a feature. An example of this analysis procedure is illustrated in Fig. 4. Various statistical values were selected from the original feature space. Because the statistical results were calculated from one column or row of a matrix, and the distribution of activations was important, the subset features included many statistical descriptors of the shape of a distribution, such as kurtosis or skewness. Figure 5 shows the averaged four NMF activation matrices based on these 15 nonstationary subset features. To observe the overall distribution of the magnitudes corresponding to each dimension-reduced cycle frequency *α* and spectral *f* index pair, we calculated the average matrices, including the 15 nonstationary subset features of all subjects, and adapted a Gaussian filter to contour the view of each matrix.

**Table 1:** Final subset features selected from the original feature set. From the original feature set that consisted of temporal analysis and nonstationary analysis features, a dimension reduction technique and feature selection method were adapted for more efficient subset feature and classification accuracy

Nonstationary analysis subset features				
Rank	Base	Observation	Statistics	Sequence number
1	*α*	*f*	Maximum	40
2	*α*	*f*	Maximum	42
3	*α*	*f*	Variance	34
4	*α*	*f*	Kurtosis	8
5	*f*	*α*	Kurtosis	42
6	*f*	*α*	Maximum	24
7	*f*	*α*	Standard deviation	42
8	*f*	*α*	Variance	24
9	*f*	*α*	Median	24
10	*f*	*α*	Median	45
11	*f*	*α*	Median	46
12	*f*	*α*	Mean	7
13	*f*	*α*	Kurtosis	1
14	*f*	*α*	Kurtosis	2
15	*f*	*α*	Skewness	2

Temporal analysis subset features	
Rank	Energy level transition information
16	(1 x 1) from Level 1 to Level 1
17	(3 x 4) from Level 3 to Level 4
18	(4 x 1) from Level 4 to Level 1

According to Fig. 5, the averaged four NMF activation matrices have different distributions with regard to OSA severity. The normal group demonstrates more widespread spectral activations associated with a dimension-reduced cycle frequency index domain than the others. The mild group’s distribution is relatively sparser than any other groups, in particular, it has no evident cyclic activations related to a low range of the spectral index. The activation distribution of the moderate group demonstrates relatively high cyclic activation at a low range of the spectral index. The severe group’s distribution demonstrates higher spectral activations at a high range of cyclic indices and higher cyclic activations at a high range of spectral indices when compared with the other groups. Moreover, the significant highest activation regions of the NMF activation matrices are all different according to the OSA severity. Based on these observations, we can assume that our nonstationary feature set is useful for classifying the breathing sounds into four OSA severity groups.

Regarding temporal analysis subset features, three features were selected from the original set and they represented the transition probability of the approximated breathing sound’s energy values. The selected transition information indicated that the silent section and those associated with a predefined OSA candidate were important for OSA severity classification in this feature set. With these temporal analysis features, we were able to perform statistical analysis to check the differences between four OSA severity classes. Using analysis of variance (ANOVA) and Tukey’s honest significance difference (HSD) test, we verified that all three temporal analysis features were significant (*p* < 0.05), and most class-pairs (normal-mild, normal-severe, normal-moderate, mild-severe, and moderate-severe), with the exception of the moderate-mild class-pair, demonstrated a significant difference (*p* < 0.05) regarding these features. The analysis results are shown in Table 2.

**Table 2:** Results of temporal analysis subset features

Temporal analysis feature		1 × 1	3 × 4	4 × 1
ANOVA		***	***	***
Tukey HSD	Moderate-mild	n/s	n/s	n/s
	Normal-mild	**	n/s	n/s
	Severe-mild	***	***	***
	Normal-moderate	***	n/s	**
	Severe-moderate	**	***	***
	Severe-normal	***	***	***

** (0.001 < p < 0.01)

*** (p < 0.001)

*n/s* not significant

### Classification test with the subset features

Using the subset features, we performed the four-OSA severity classification test. We trained the classification model using an SVM [25–27] with a linear kernel and confirmed the model’s performance using LOOCV. All the experiments were conducted using a WEKA-implemented classifier and validation tools, and their configuration settings initialized with default values. Also, in order to select the best classifier, classification experiments were performed using various classifiers built in WEKA framework such as random forest, bayes network, logistics and the SVM was finally selected among them. The detailed results of cross-validation are shown in Table 3. The moderate OSA group has the lowest true positive (TP) rate, while the severe group has the highest TP rate.

**Table 3:** Detailed results of cross-validation

Group	True positive rate	False positive rate	Precision	Recall	F-measure	ROC area	PRC area
Normal	0.75	0.02	0.94	0.75	0.83	0.93	0.81
Mild OSA	0.86	0.16	0.64	0.86	0.74	0.81	0.59
Moderate OSA	0.67	0.08	0.74	0.67	0.70	0.77	0.58
Severe OSA	0.91	0.02	0.95	0.91	0.93	0.98	0.92
Weighted average	0.80	0.07	0.82	0.80	0.80	0.87	0.72

The classification accuracy of the four-OSA severity classification test was 79.52%. Table 4 shows the classification result as a confusion matrix. In the moderate OSA group, it showed classification errors with respect to the mild OSA group. Moreover, the majority of the normal subject group’s classification error is related to the mild OSA group. Using Table 4, we can also obtain the binary classification result to classify normal subjects (AHI ≤ 5) and OSA patients (AHI ≥ 5). The binary classification results show that the sensitivity is 98.0%; specifically, it is 75.0% and the classification accuracy is 92.78%.

**Table 4:** Four-OSA severity classification result with leave-one-out cross-validation

	Classified as			
	Normal	Mild OSA	Moderate OSA	Severe OSA
Normal	15	4	1	0
Mild OSA	1	18	2	0
Moderate OSA	0	6	14	1
Severe OSA	0	0	2	19

With reference to the comparison proposed in a related study [12], in Table 5, we compared our method with other studies in terms of the number of subjects, microphone’s location, number of OSA groups, and performance.

**Table 5:** Method comparison between related studies using snoring sounds

Method	Subjects	Microphone’s location	Number of OSA groups	Sensitivity	Specificity
				Accuracy (%)	
Nakano [7]	383	Neck (contact)	Two	93	67
Abeyrantne [8]	16	Patient vicinity (40–70 cm)	Two	100	50
Azarbarzin [12]	57	Neck (contact)	Two	92.9	100
			Four	77.2	
Behar [13]	856	Face (contact)	Two	69.5	83.7
Proposed	83	Patient vicinity (170 cm)	Two	98.0	75.0
			Four	79.52	

Since there is no standardized performance comparison framework for studies using sleep breathing sounds [12], our study may not be evaluated as being objectively superior. However, this table is presented to check our research performance level against the previous related studies.

## Discussion

In this study, we have demonstrated that our new sleep breathing sound analysis method can provide relatively high performance for multiple OSA severity classification. We hypothesized that the energy transitions related to the general breathing sounds, snores, and silence in a time series, and the cyclostationarity-based nonstationary characteristics of the sounds associated with obstruction and vibration in the upper airway could be used as significant features that represent a full night of breathing sounds of a subject. This hypothesis was tested using experiments that classified the sleep breathing sounds into OSA severity classes based on the AHI.

In this study, we extracted nonstationary features using an entirely new approach. We calculated an average CSD from a subject’s sleep breathing sounds, for which the average time was over 4 h. Then the NMF method was adapted for drastic dimension reduction, and a feature selection strategy was also applied to search core subset features. This is the first attempt at this approach for breathing sound analysis. The results of this method show that cyclostationary activation, which represents a hidden periodicity of data related to particular spectral bands, could be a special feature for sleep breathing sounds. Using this approach, we summarized all the nocturnal breathing sounds of each subject, with particular properties that were associated with the spectral characteristics and hidden periodicity of the sounds. We verified that this feature represented significant differences between breathing sounds, which were grouped according to the OSA severity class, as shown in Fig. 5. For the normal group, the NMF activation matrix showed that a wide spectral band area was associated with the narrow high indexed cycle frequency band area. By contrast, the moderate and severe OSA groups presented different characteristics. For these groups, a wide cycle frequency band area associated with a particularly high indexed spectral area was activated in the matrix. We found that these properties reflected the special spectral characteristics of representative breathing sounds of each subject’s nocturnal breathing sounds.

The temporal analysis features were the transition probability of breathing sound energy in the time series. We adapted basic OSA detection criteria that are related to the silent interval between snoring sounds, for example, apneic events greater than 10 s in duration [4]. In Table 1, the final temporal analysis subset feature consisted of three types of transition information: (1 × 1)—silence level (3x4)—energy transition from the high energy level to the OSA candidate level, and (4x1)—energy transition from the OSA candidate level to the silence. We showed that all the features were statistically significant in Table 2 in which the appearance rates of purely silent sections and the sections of energy transition to silence could be a significant feature for the OSA severity classification task.

The aforementioned two features could be influenced by the sound recording quality with respect to the recording performance or location of the microphone. In this study, we used an ordinary microphone that was not specifically installed to record or analysis breathing sounds and was located far away from the subject (almost 2 m); thus, we judged that the breathing sounds used in our experiments already had a low sound recording quality. In a typical experiment or real application, the recording equipment will be located near the user, so the recording quality will be similar or better than the sound used in our experiment. Therefore, we expect our features to perform well in an individual space, such as private bedroom with an ordinary sound recording device.

In Table 5, we compared our study with previous research. Nakano et al. [7] recorded the tracheal sound using a body-contact microphone and calculated a transient fall (TS-dip) of the power spectra’s moving average in the time series. With this feature, they obtained the tracheal sound-respiratory disturbance index (i.e., the number of TS-dips per hour) and compared it with existing AHI values from PSG. The result of OSA subject detection using their feature (AHI threshold 5) was 93% sensitivity and 67% specificity. Abeyratne et al. [8] detected segments of snore-related sounds (SRS) detected automatically and categorized SRS into pure breathing, silence, and voiced/unvoiced snoring segments. From these segments, they extracted the intra-snore pitch periods feature, which was characterized by discontinuities called intra-snore-pitch-jumps. Using this feature, they obtained an OSA detection result with 100% sensitivity and 50% specificity, where the AHI threshold was 5. Azarbarzin et al. [12] recorded sleep breathing sounds with a special microphone that was placed over the suprasternal notch of the trachea, and extracted three types of segment: non-apneic, hypopneic, and post-apneic. From these segments, they calculated the total variation norms of the zero crossing rate and peak frequency, and used them as features. They obtained 77.2% accuracy from the four-OSA severity classification test and an additional OSA detection result of 92.9% sensitivity and 100% specificity with AHI threshold 5. Behar et al. [13] detected OSA subjects using breathing sounds and additional information from sensors such as actigraphy, body position assessment, and photoplethysmography. For breathing sound recording, they used a particular microphone attached to the subject’s face, and extracted the multiscale entropy values from the audio. The OSA subject detection result based on audio was 69.5% sensitivity and 83.7% specificity for training using SVM. Unlike previous studies, our proposed method did not use a special body contact-type microphone and did not perform any snoring segmentation with breathing sounds recorded at long distance. We only divided the sleep breathing sound into predefined window lengths and generated the feature matrix using the aforementioned two features from all the windows. Because we intend to use this method for a screening test system that can provide information on OSA risk or notification for a PSG test for individuals operated using a personal mobile device, we suppose that our proposed method, environment setup, and result are suitable for our purpose.

## Conclusions

In this study, we proposed an OSA severity classification technique for a preliminary PSG test using particular features of nocturnal sleep breathing sounds. Unlike recent studies, this research did not use any of the conventional features that were used in existing sound analysis domain. Instead, we used only the audio signal’s energy transition probability information in the time domain, and the cyclostationarity-based nonstationary characteristics in the spectral and cycle frequency domains. Using these two features, the proposed method showed the most competitive classification performance of 79.52% for the four OSA severity classification and 92.78% for OSA patient detection test. These results indicate that a proposed method could be a promising approach to identify the multiple OSA severity of suspected patients and provide proper information to individuals for a preliminary PSG screening test.

The limitations of conventional PSG, such as the high cost, inconvenience, complex measurement method, and difficulty of ensuring sleep variability because of the single night’s test, lead to an increased demand for a preliminary PSG screening test in various environments, such as the home. In the proposed method, the sounds were recorded only in the clinical test rooms, so it can be considered that the experiment did not consider the actual environments. However, since the general private bedroom environment is not much different from the PSG room, and the sounds were not recorded under particular controlled conditions, the proposed algorithm is expected to perform reasonably well in universal circumstances.

The proposed method has some limitations. For more practical applications, there is a need to apply various noise reduction and cancellation techniques to the acquired sounds or framework of research. Additionally, experiments with many more patients should be conducted to make our method more robust and reliable. Furthermore, to obtain more accurate classification performance, additional algorithms can be considered in the preprocessing or feature extraction step. In particular, the properties related to cyclostationarity can be good input data of feature learning techniques for a deep neural network; thus, we will consider this in a future study.

The present study will contribute to the development of screening technology for a specific medical inspection using restricted data, and we expect that this technique will be applied within various mobile healthcare platforms to supplement a preliminary home examination of sleep disorders.

## Abbreviations

PSG: polysomnography
OSA: obstructive sleep apnea
AHI: apnea hypopnea index
AASM: American association of sleep medicine
NREM: non-rapid eye movement
CSD: cyclic spectral density
NMF: non-negative matrix factorization
*rmsS*_*xx*_*(α,f)*: real-time mean of CSD
RMS: root mean squared
SVM: support vector machine
LOOCV: leave-one-out cross-validation
ANOVA: analysis of variance
HSD: honest significance difference
TP: true positive
